# Strong localization effect and carrier relaxation dynamics in self-assembled InGaN quantum dots emitting in the green

**DOI:** 10.1186/s11671-015-0772-z

**Published:** 2015-02-03

**Authors:** Guo-En Weng, Wan-Ru Zhao, Shao-Qiang Chen, Hidefumi Akiyama, Zeng-Cheng Li, Jian-Ping Liu, Bao-Ping Zhang

**Affiliations:** Department of Physics and Semiconductor Photonics Research Center, Xiamen University, 422 South Siming Road, Xiamen, 361005 P. R. China; Department of Electronic Engineering, Optoelectronics Engineering Research Center, Xiamen University, 422 South Siming Road, Xiamen, 361005 P. R. China; Institute for Solid State Physics, The University of Tokyo, 5-1-5 Kashiwanoha, Kashiwa, Chiba 277-8581 Japan; Department of Electronic Engineering, East China Normal University, 500 Dongchuan Road, Shanghai, 200241 P. R. China; Suzhou Institute of Nano-Tech and Nano-Bionics, Chinese Academy of Sciences, 398 Ruoshui Road, Suzhou, 215123 P. R. China

**Keywords:** InGaN, Quantum dots, Localization effect, Carrier relaxation dynamics

## Abstract

Strong localization effect in self-assembled InGaN quantum dots (QDs) grown by metalorganic chemical vapor deposition has been evidenced by temperature-dependent photoluminescence (PL) at different excitation power. The integrated emission intensity increases gradually in the range from 30 to 160 K and then decreases with a further increase in temperature at high excitation intensity, while this phenomenon disappeared at low excitation intensity. Under high excitation, about 40% emission enhancement at 160 K compared to that at low temperature, as well as a higher internal quantum efficiency (IQE) of 41.1%, was observed. A strong localization model is proposed to describe the possible processes of carrier transport, relaxation, and recombination. Using this model, the evolution of excitation-power-dependent emission intensity, shift of peak energy, and linewidth variation with elevating temperature is well explained. Finally, two-component decays of time-resolved PL (TRPL) with various excitation intensities are observed and analyzed with the biexponential model, which enables us to further understand the carrier relaxation dynamics in the InGaN QDs.

## Background

Ш-nitride-based wurtzite semiconductors InN, GaN, AlN, and their alloys have attracted considerable attention in recent years due to their promising applications in solid state lighting, high-density optical storage, and full-color display [1-3]. Depending on the alloy composition, Al_*x*_In_*y*_Ga_*z*_N systems are in principle able to cover a wide spectral range from ultraviolet to near infrared [4]. Although the developments of the crystal growth technology have led to the commercialization of dazzling blue and green InGaN quantum well (QW) light-emitting-diodes (LEDs) and laser diodes (LDs), the so called “green gap” that the internal quantum efficiency (IQE) of InGaN QW drops significantly when going to green and longer wavelength regions is still a devilish problem to solve, which is ascribed to 1) increased spacial separation of electron-hole wave function induced by the stronger polarization field with increasing strain [5,6] and 2) the degraded material quality caused by the increased lattice mismatch between high-In-content InGaN and GaN [7]. In order to circumvent this problem inherent in InGaN QW active layers, an alternative InGaN quantum dot (QD) structure has been suggested as light-emitters in the green or longer spectral ranges. It has been shown both theoretically [8,9] and experimentally [10,11] that the piezoelectric polarization field and resulting quantum-confined Stark effect (QCSE) in InGaN QDs are much smaller than in QWs. Moreover, InGaN QDs inherently contain a lower density of structural defects due to the build-in strain field, leading to a reduced efficiency droop and a higher brightness [11,12].

In the last 2 decades, growth techniques of InGaN QDs, i.e., metalorganic chemical vapor deposition (MOCVD) and molecular-beam epitaxy (MBE), are extensively studied and well developed. By using these methods, InGaN QD LEDs and LDs, which emit from green to red, have recently been demonstrated with superior performance over equivalent QW-based counterparts [13-17]. However, only few optical investigations on the carrier transport and relaxation in InGaN QDs have been made to further understand the carrier recombination processes [18-20]. As is well known, localization effects are discovered in blue InGaN QWs with clear physical model of carrier transport among localized states [21]. In the InGaN QDs, nevertheless, since the higher indium content, the localized luminescence centers must be much more deeper than that in QWs, which influences the carrier transport, relaxation, and recombination processes in the QDs. In this letter, strong localization effect in self-assembled InGaN QDs is evidenced by temperature (*T*)-dependent photoluminescence (PL) under different excitation power (*P*). The evolution of excitation-power-dependent emission intensity, shift of peak energy, and linewidth variation with elevating temperature is analyzed using a strong localization model. Additionally, time-resolved PL (TRPL) measurements are performed to further understand the carrier relaxation dynamics in the InGaN QDs.

## Methods

**Figure 1:**
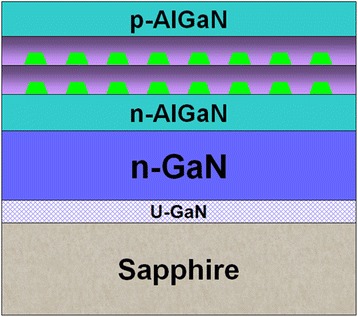
**Schematic diagram of the QD epitaxial structure.**

The InGaN self-assembled QD sample investigated in this work was epitaxially grown on a (0001)-oriented sapphire by MOCVD system [22]. A schematic diagram of the QD epitaxial structure is shown in Figure 1. The active region consisted of two pairs of InGaN/GaN QDs. The GaN cap layers on QDs were deposited using a two-step method: firstly, a 2-nm-thick low-temperature grown GaN matrix layer was deposited at the same growth temperature (670°C) of QDs to protect them during subsequent temperature ramping process, then the temperature was ramped to 850°C, and finally, a 8-nm-thick GaN barrier layer was grown. The indium content of the QDs is about 27%. Other detailed growth procedures are available in ref. [22]. Cross-section Z-contrast scanning transmission electron microscopy (STEM) shows that the diameters of the QDs range from 20 to 60 nm, while the average height of QDs is about 2.5 nm.

Temperature-dependent PL measurements were carried out using a Q-switched YVO_4_ pulse laser emitting at 355 nm with a pulse width of 25 ns and a repetition rate of 30 kHz. The temperature of the sample was controlled within a closed-cycle helium refrigerator. The PL signal was dispersed with a triple-grating spectrometer and detected with a liquid-nitrogen-cooled charge-coupled device. For TRPL experiments, the measurement was performed by a fs impulsive optical excitation at 400 nm under various excitation densities. The 400-nm pulses were generated from a mode-locked Ti:sapphire regenerative amplifier system operating at 150-fs pulse duration and 1-kHz repetition rate (Spitfire, Spectra-Physics, Newport Corporation, Irvine, USA). Time traces of amplified spontaneous emission (ASE) of the sample were characterized with a streak camera system with a temporal resolution of about 20 ps.

## Results and discussion

**Figure 2:**
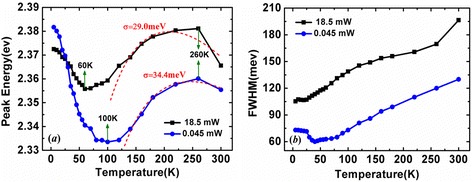
**Temperature dependence of emission peak energies (a) and spectra linewidths (FWHM) (b) measured under different excitation power.** The dashed lines are the fitting results using Varshni’s model.

The temperature dependences of PL experiments were performed on the QD sample over a temperature range of 5 to 300 K under different excitation power: 0.045 and 18.5 mW, respectively. By fitting the PL spectra with a Gauss peak, the emission peak energies, spectral linewidths [full width at half maximum (FWHM)], and emission intensities are determined. As shown in Figure 2a, both the peak energies (*E*_*p*_) show an “S shape” (decrease-increase-decrease) variation with increasing temperature. Although this emission behavior is somewhat similar to those reported in InGaN QWs, the temperature of the turning point from blueshift to redshift (*T* = 260 K) is found much more higher, which is believed to be relevant to the more deeper localization potential in QDs. It is found that the S-shape variation behavior depends on excitation intensity. Under high excitation intensity (*P* = 18.5 mW), *E*_*p*_ is initially smaller (*T* = 5 K) and then quickly becomes larger at elevated temperature (*T* > 30 K), meaning a narrower variation range of the peak position in comparison with that under weak excitation *(P =* 0.045 mW*).* Another dissimilarity is that the blueshift starts at a lower temperature of 60 K for the high excitation. As discussed later, these behaviors are ascribed to the distinct carrier redistribution in localized state assemblies with increasing temperature.

Figure 2b plots the temperature-dependent PL linewidth at different excitation power investigated. For the power of 0.045 mW, the linewidth exhibits a slight reduction followed by a linearly increment up to 300 K. However, the linewidth with *P =* 18.5 mW shows a continuous increase over the entire temperature range. Furthermore, a considerable broadening of the emission linewidth is also observed since the band-filling of the localized states under high excitation intensity.

**Figure 3:**
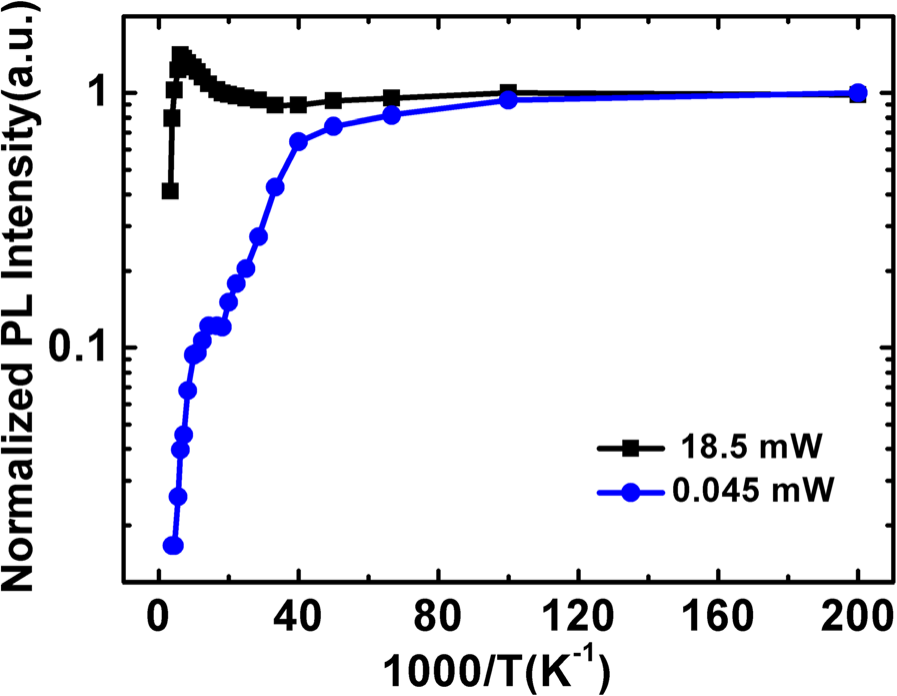
**Normalized integrated PL intensity as a function of 1/** ***T*** **for the InGaN QD emission.**

The integrated emission intensities of the QD sample are given in Figure 3 as a function of the reciprocal of temperature. The temperature-dependent emission intensities were normalized by the integrated emission intensity at 5 K. It is quite significant to notice that an anomalous enhanced emission appears over a temperature range of 30 to 160 K at high excitation intensity. However, for the weak excitation, the emission intensity decreases monotonously with a small “uplift” in the same temperature range and then decreases more rapidly with further increase of the temperature. In the former case, the maximum emission intensity at 160 K is enhanced by 40% in comparison to that at low temperature. Beside, a high internal quantum efficiency (IQE) of approximately 41% is obtained by the ratio of emission intensity at room temperature and that at 5 K, assuming that the radiative recombination is dominant at sufficiently low temperature.

**Figure 4:**
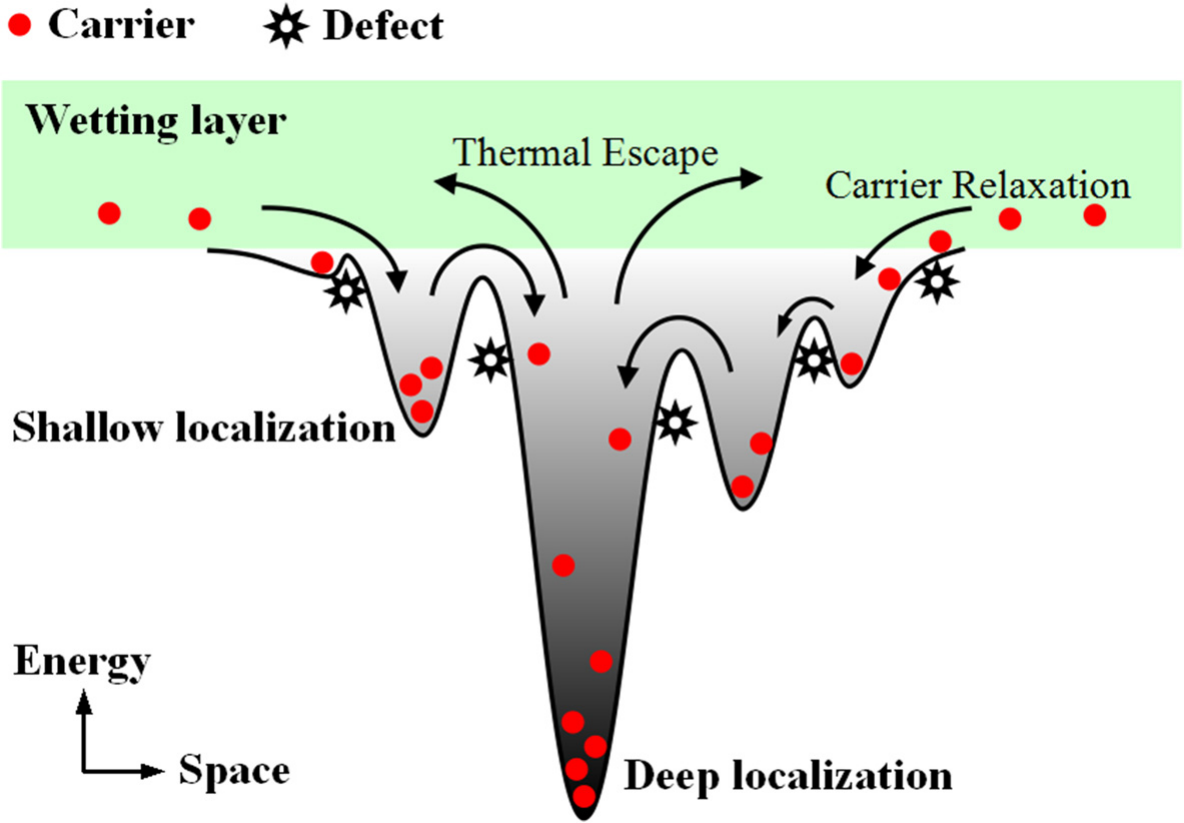
**Schematic diagram of potential distribution of the non-isolated QDs describing possible processes of carrier transport, relaxation and recombination.**

**Figure 5:**
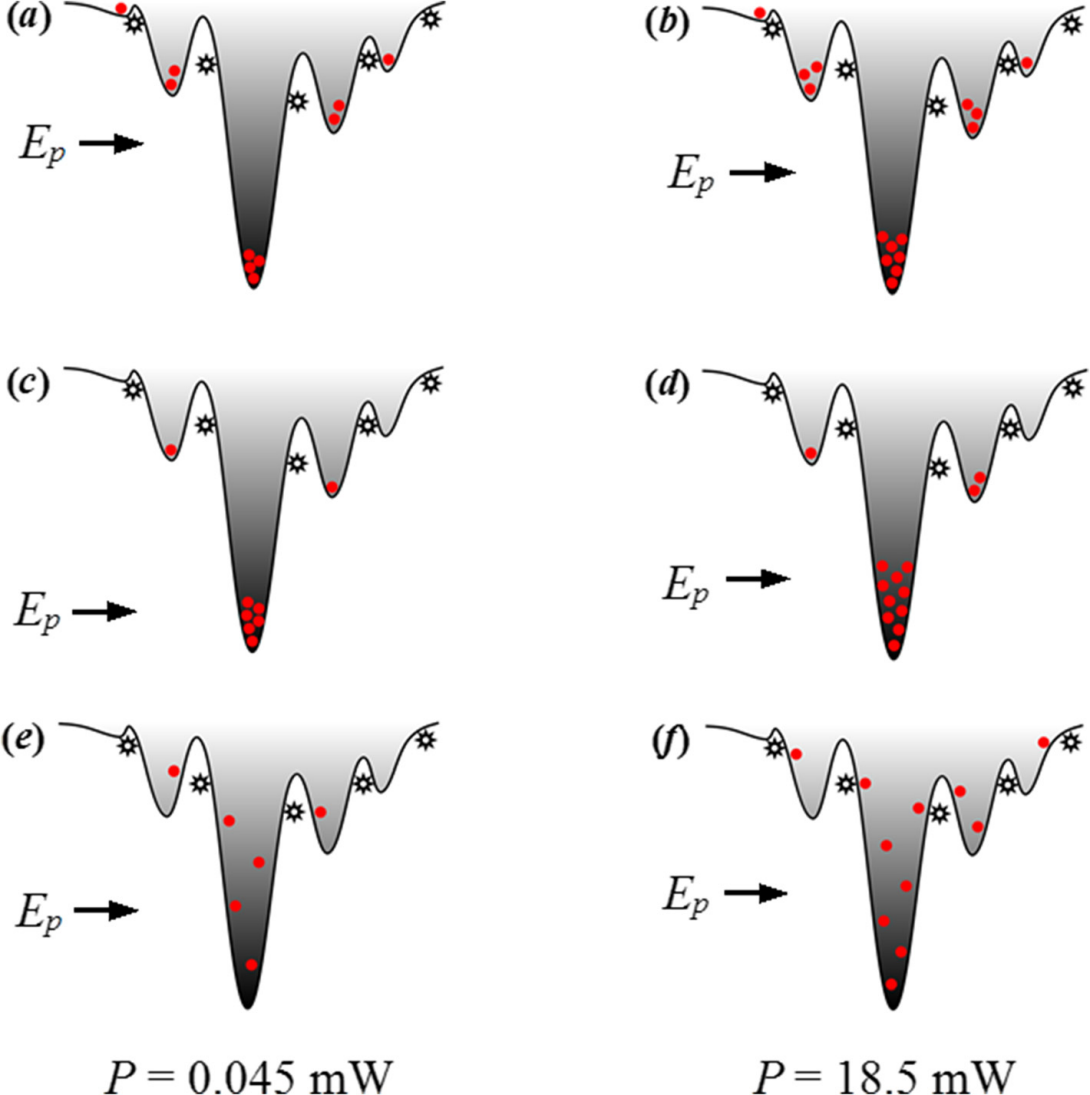
**Schematic diagrams indicating the possible mechanism of the S-shaped temperature dependent PL peak energy, linewidth evolution, and anomalous emission enhancement.** The carrier distributions at lowest *T* (5 K), temperature that starts to blueshift (60/100 K) and room temperature are shown in **(** ***a*** **)**, **(** ***c*** **)**, **(** ***e*** **)** and **(** ***b*** **)**, **(** ***d*** **)**, **(** ***f*** **)** for *P* = 0.045 and 18.5 mW, respectively.

To explain the observed different variation under different excitation power in 1) the S-shaped temperature-dependent emission energy, 2) the evolution of linewidth as well as 3) the variation of emission intensity, a strong localization model describing possible processes of carrier transport, relaxation, and recombination is proposed, as shown in Figure 4. In comparison with the localization model in blue InGaN QWs, the localized luminescence centers in green InGaN QDs are much more deeper for the higher indium content and have stronger quantum confinement effect (QCE). Using this model, the distinct phenomena observed in our experiments can be well explained as follows: At low temperature of 5 K, carriers are randomly distributed among both deep and shallow potential minima caused by potential fluctuations and the radiative recombination process is dominant. For high excitation intensity (*P* = 18.5 mW), the carrier concentration is much higher and, as a result, a majority of photo-generated carriers are trapped in deep localization states, resulting in a smaller *E*_*p*_ [Figure 5b]. As the temperature increases from 5 to 60 K (100 K), shallow localized carriers are thermally activated and relax down into other deep localization via hopping, leading to the initial redshift of *E*_*p*_ as large as 16 meV. This is also true for low excitation case, but the redshift is much larger, 48 meV. The relatively smaller decrement in *E*_*p*_ for the high excitation is ascribed to the more markedly band filling effect in the deep localization centers. In case of low excitation where the carrier density is much lower, on the other hand, most of the carriers can relax into the lowest energy level of the deep localization center with rising in temperature up to 100 K, and thus, the carrier distribution narrows [Figure 5c], accompanied by a remarkable redshift of *E*_*p*_ and a slightly decrease of the PL linewidth [Figure 2]. When further increase of the temperature above 60 K (100 K), band-filling in the deep localization is dramatically enhanced due to the temperature-dependent intra-dot relaxation behavior of the carriers. In addition, the regular thermalization of the carriers becomes more and more significant. These temperature-dependent thermal broadening effects of the carriers lead to a continuous increase of the spectra linewidths for both excitation intensities, as shown in Figure 2b. The anomalous enhanced emission over the temperature range of 30 to 160 K at high excitation intensity observed in Figure 3 can be understood as the following. As is well known, with the temperature increases, the defect-related non-radiative recombination would be more and more serious and then results in the quenching of PL. In fact, however, the emission intensity increases monotonously in this temperature range despite of the aggravated non-radiative recombination, which is also observed by Ma et al. [20] and Masumoto and Takagahara [23]. Due to the stronger QCE and better crystal quality in deep localization, it is convincing to consider that the radiation efficiency of carriers in deep localization is higher and thus resulting in a faster increase in emission intensity. With elevating the temperature, the carriers escape from the shallow localization and then converge to fill in the deep localization center by relaxation and recapture processes. Hence, the carriers consumed by radiative recombination in deep localization can be compensated rapidly. As a consequence, a majority of carriers would radiate in the deep localization with a higher radiation efficiency and eventually lead to an anomalous enhanced emission. The maximum emission intensity at 160 K is enhanced by 40% in comparison to that at low temperature, and a high IQE of approximately 41% is obtained at room temperature. For weak excitation, however, the carrier density is much lower. The defect-assisted non-radiative recombination should not be disregarded anymore with rising temperature. During the relaxation and recapture processes, a considerable proportion of carriers would be captured by the non-radiative centers, which suppresses the compensation of carriers that consumed by radiative recombination and results in a monotonous decreasing of the emission intensity. It is rather complicated but reasonable for the emission intensity that decreases slower with a small “uplift” in this particular temperature range, as balanced with the improved radiation efficiency in the deep localization and the aggravated non-radiative recombination during the relaxation or recapture processes. For *T* > 160 K, the carriers may have sufficient energy to repopulate the shallow localization. The non-radiative recombination then gradually dominates the recombination process, leading to a rapid quenching of PL. On the other hand, the thermal-induced band-filling and carrier redistribution contribute to a conspicuous blueshift of the *E*_*p*_ [Figure 2a]. At even higher temperature, most carriers start to escape from the localized states and become free carriers. A redshift of the *E*_*p*_ is then observed due to the temperature-induced bandgap shrinkage. It should be noted that the temperature of the turning point from blueshift to redshift is as high as 260 K. Such high temperature denotes that the carriers need large energy to escape from the deep localization, indicating a strong confinement effect in the localization potentials. Such temperature-dependent emission energy at localized states can be described as [24]:

1 $$ E(T)=E(0)-\frac{\alpha {T}^2}{T+\beta }-\frac{\sigma^2}{k_BT}, $$

where *E*(0) is the energy gap at 0 K, *α* and *β* are the Varshni coefficients, *σ* indicates the degree of the localization effect, and *k*_*B*_ is the Boltzmann constant. Using this formula to fit the data, see Figure 2a, we obtain *σ* to be 34.4 and 29.0 meV for weak and strong excitation, respectively. Both the values of *σ* are found to be more larger than that in the QWs [25], meaning a much stronger localization effect in the QDs.

**Figure 6:**
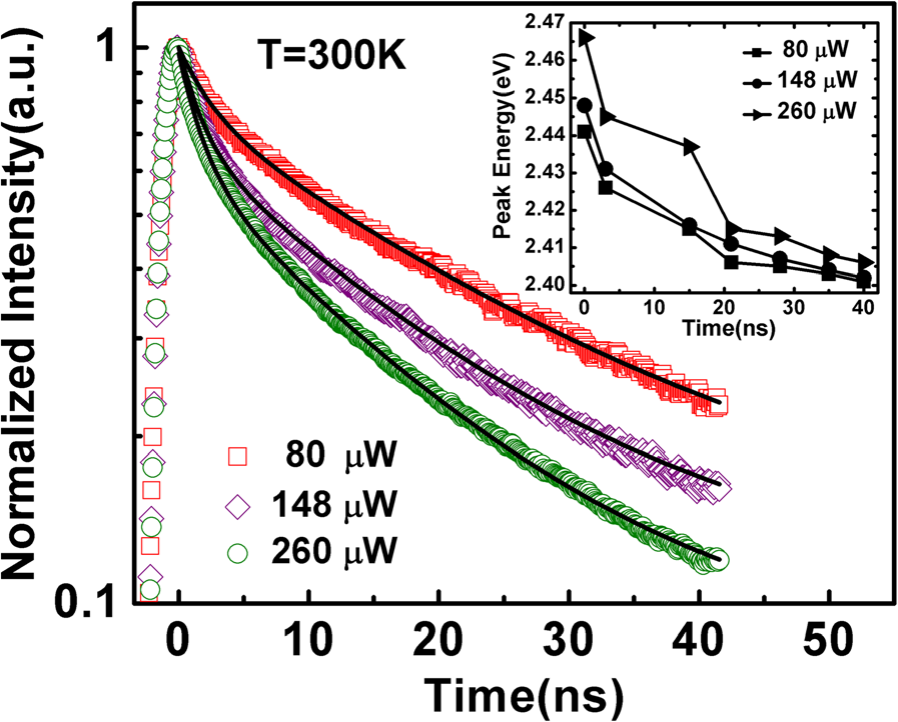
**TRPL decay curves of the QDs at different excitation power.** The solid curves are biexponential fits to experiment data. The inset shows temporal variation of the peak energy of the PL spectra.

In order to further clarify the carrier transfer process and relaxation dynamics of the InGaN QDs, TRPL measurements are performed by fs impulsive excitation at room temperature. As shown in Figure 6, the normalized TRPL decay curves with varied excitation densities exhibit two obvious decay stages, which are relatively faster in the early stage and slower in the extended range. The decay curves can be well fitted with a biexponential function [26]:

2 $$ I(t)={B}_1{e}^{-t/{\tau}_1}+{B}_2{e}^{-t/{\tau}_2}, $$

where *τ*_*1*_ and *τ*_*2*_ represent the carrier lifetime in the fast and slow decay stages, respectively. The obtained fitting results are listed in Table 1.

**Table 1:** **Carrier lifetime in the fast and slow decay stage at different excitation power**

**Power (μW)**	***τ***_**1**_ **(ns)**	***τ***_**2**_ **(ns)**
80	2.5	23.1
148	1.8	18.1
260	1.7	15.9

**Figure 7:**
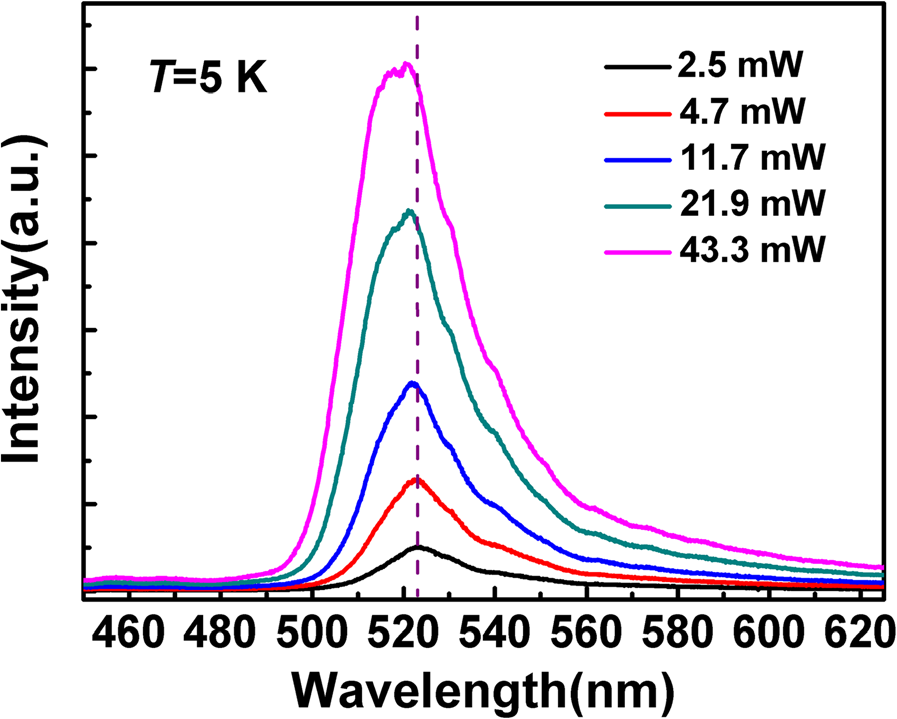
**Excitation power dependence of PL spectra.**

According to the schematic diagram indicating possible paths of carrier transport and relaxation shown in Figure 4, we can give a reasonable explanation of the two decay stages and make a better understanding of the carrier dynamics in the InGaN QDs. Just after the pulse excitation, where the carrier density is high, abundant high-energy carriers in the weakly localized states recombine rapidly. Moreover, fast carrier outflow on the high-energy side, carrier relaxation from weakly to strongly localized states, and serious non-radiative recombination of high-energy carriers should also be considered [27]. All these carrier behaviors act simultaneously and hence contribute to the fast early-stage decay, which is more remarkable at higher excitation power. With the time going on, most of the remaining high-energy carriers would be recaptured by the deeply localized states, which results in a longer lifetime because of the stronger localization effects. Such an argument is consistent with the result that the PL peak energy redshifts fast in the early-stage and then slow down, as shown in the inset of Figure 6. The spectral intensity decays slower on the low-energy side in comparison with that on the high-energy side. Note that the well-known QCSE and the carrier screening effect in the InGaN system play important roles on carrier lifetime and transition strength. As Kuroda and Tackeuchi [28] reported, since the carrier density decreases over time due to recombination, the screening of the QCSE becomes weaker. Then, the increase of spatial separation between electrons and holes causes a decrease in the recombination rate, resulting in a longer lifetime. Nevertheless, in our experiments, the excitation power-dependent PL spectra shown in Figure 7 exhibit a slight blueshift of the PL peak position together with the broadening of spectra linewidth at shorter wavelength. Such line shape evolution and slight blueshift of the peak energy are exactly caused by the band-filling effect rather than QCSE. It demonstrates that the QCSE in the investigated InGaN QDs is very small and can be ignored. Consequently, we attribute the carrier emission behaviors and carrier lifetimes discussed above to the strong localization effect during the carrier transport and recombination processes in the InGaN QDs that with high indium content.

## Conclusions

In this study, we have investigated the carrier relaxation dynamics of the green-emitting InGaN QDs over the temperature rang of 5 to 300 K at different excitation power. The temperature-dependent S-shaped peak-energy shift and linewidth evolution reflect the strong localization effect and carrier transfer processes in the QDs. For high excitation, about 40% emission enhancement at 160 K and a high IQE of 41.1% are obtained, which arises from the effective carrier filling of the deeper localization potentials that having higher radiation efficiency. The TRPL decay curves with varied excitation densities exhibit two obvious decay stages, which are relatively faster in the early stage and slower in the extended range, corresponding to the high-energy carrier recombination, overflow, escape, relaxation, and the recapture behavior of the deep localization.

## Abbreviations

QDs: Quantum dots
PL: Photoluminescence
IQE: Internal quantum efficiency
TRPL: Time-resolved photoluminescence
QW: Quantum well
LEDs: Light emitting diodes
LDs: Laser diodes
QCSE: Quantum-confined Stark effect
MOCVD: Metalorganic chemical vapor deposition
MBE: Molecular-beam epitaxy
STEM: Scanning transmission electron microscopy
ASE: Amplified spontaneous emission
FWHM: Full width at half maximum
QCE: Quantum confinement effect
